# A Comprehensive Approach to Clinical Staging of Bladder Cancer

**DOI:** 10.3390/jcm11030761

**Published:** 2022-01-30

**Authors:** Przemysław Adamczyk, Paweł Pobłocki, Mateusz Kadlubowski, Adam Ostrowski, Andrzej Wróbel, Witold Mikołajczak, Jan Adamowicz, Tomasz Drewa, Kajetan Juszczak

**Affiliations:** 1 Department of General and Oncologic Urology, Nicolaus Copernicus Hospital in Torun, 87-100 Torun, Poland; poblocki@gmail.com (P.P.); kadlub1@o2.pl (M.K.); witoldmikolajczak18@gmail.com (W.M.); t.drewa@wp.pl (T.D.); kaj.juszczak@gmail.com (K.J.); 2 Clinic of General and Oncologic Urology, Collegium Medicum, Nicolaus Copernicus University, 85-067 Bydgoszcz, Poland; adostro@gmail.com (A.O.); adamowicz.jz@gmail.com (J.A.); 3 Second Department of Gynecology, Medical University of Lublin, Jaczewskiego 8, 20-090 Lublin, Poland; wrobelandrzej@yahoo.com

**Keywords:** bladder cancer, hydronephrosis, radical cystectomy, staging, Urothelial cell carcinoma

## Abstract

Background: A significant number of patients with advanced urothelial cell carcinoma are under- or over-staged. Implementation of clinical variables could be useful for improving the accuracy of clinical staging. Aim: To explore the differences between clinical and pathological diagnosis in patients with UCC, and to identify clinical variables that might play a role in under- or overstating. Materials: A total of 553 patients after radical cystectomy were included in the analysis. Clinical stage of the disease was diagnosed according to CT or MRI in relation to clinical data. Results: Higher clinical stage correlated with a higher pathological stage (*p* < 0.00005), but in 306 patients did not correspond (142 patients were under-staged and 164 over-staged). Over half (54.2%) of the patients staged as cT1–cT2 were misdiagnosed: 137 patients were under-staged and 133 over-staged. Hydronephrosis was associated with a higher pathological stage (*p* < 0.000005), mostly pT3–4 (45.13% had pT4 disease) and higher risk of nodal metastasis (*p* = 0.0028). The highest percentage of PSM was found in patients with pT4 (33.12%). Conclusions: Clinical staging of bladder cancer is poorly executed, with one third of patients under-staged and one third over-staged. To improve accuracy, we recommend a multimodal approach, combining histopathological evaluation with results of imaging studies.

## 1. Introduction

The Urothelial cell carcinoma (UCC) is the most common neoplasm of the urinary tract, with 380,000 new cases reported each year worldwide [1,2]. Currently, the Tumor Nodes Metastasis (TNM) scale is commonly used to describe the extent of the spread of UCC. Generally, patients are staged before treatment, termed the clinical or pre-treatment (cTNM) stage, and then undergo post-surgical histopathological estimation (pTNM) [3,4]. The cTNM stage (which is based on examination, imaging, and transurethral resection of the tumor with biopsy) aims to provide a clear view of the extent of the disease in order to guide primary treatment, while the pTNM assessment provides precise data for estimating prognosis and planning further treatment.

Ultrasonography (USG), computed tomography (CT), and magnetic resonance imaging (MRI) are the mainstay investigations in the pre-operative work-up of patients with UCC to establish extent and the size of the primary tumor, as well as to determine the presence and location of lymph node and/or distant organ metastasis. Subsequently, the pathological (pTNM) stage should provide information about the extent of the tumor in terms of local invasion, surgical margins, extension of lymphadenectomy, the location and number of metastatic nodes, and the extracapsular spread of the tumor [5]. However, accurate clinical staging remains challenging, and the risk of both improper staging and grading (and subsequent inadequate treatment) is considerable, but for a trained histopathologist the diagnosis of low-grade and high-grade disease is not a major problem. Indeed, the current methods of clinical assessment are subject to inter- and intra-observer errors. The commonly used imaging methods (MRI, CT) also tend to diagnose only locally-advanced disease, which is insufficient for the implementation of conservative treatment [6]. To overcome this challenge, we hypothesize that a multimodal approach to the clinical staging of UCC is needed to eliminate bias and to help to stratify patients into specific risk categories for achieving optimal outcomes while avoiding overtreatment. Thus, in this study we explored the differences between the clinical and pathological diagnosis in a series of patients with UCC. We also aimed to investigate the clinical variables that might play a role in under- or overstating the extent of the disease.

## 2. Materials and Methods

### 2.1. Patients

In this retrospective study, we analyzed the data from 533 consecutive patients from three institutions who underwent radical cystectomy for the treatment of invasive UCC between 2012 and 2020. Indications for radical cystectomy, in accordance with the guidelines of the European Association of Urology (EAU), were as follows: urothelial cT2–cT4a disease or non-invasive papillary cancer that could not be controlled by transurethral resection of the bladder tumor [7]. Neoadjuvant chemotherapy was administered according to the decision of the multidisciplinary team. During radical cystectomy, which included the resection of prostate in men and of the reproductive system in woman, the obturator, external, internal, common iliac, and presacral lymph nodes were dissected for pathological analysis according to previously described procedures [8]. All patients underwent a preoperative examination, which included routine laboratory tests, a chest radiogram, abdominal USG, and a CT or MRI. For those who received neo-adjuvant chemotherapy, re-staging CT was performed and used for final evaluation.

Oncological variables and results were noted, and neoplasm staging was performed according to the TNM classification system of the Union Internationale Contre le Cancer [9]. All perioperative data were analyzed according to these variables.

### 2.2. Ethical Considerations

The study was a retrospective chart review, and informed consent and all experimental protocols were approved by Komisja Bioetyczna Collegium Medicum w Bydgoszczy (KB439/2013). All procedures performed were in accordance with the ethical standards of the local ethics committee and with the Declaration of Helsinki (1964) and its later amendments, or comparable ethical standards.

### 2.3. Statistical Analysis

The Pearson chi-squared test was used to analyze relationships between qualitative variables, such as the pTNM stage, the cTNM stage, presence of hydronephrosis, and the positive surgical margin ratio. Frequency distributions are presented in the contingency tables. The relationship between hydronephrosis and the number of metastatic nodes was based on an analysis of variance. The results were expressed as mean and standard deviation. Statistical significance was considered at *p* ≤ 0.05 for all tests.

## 3. Results

Basic clinical-pathological characteristics of study group are presented in Table 1.

**Figure 1:**
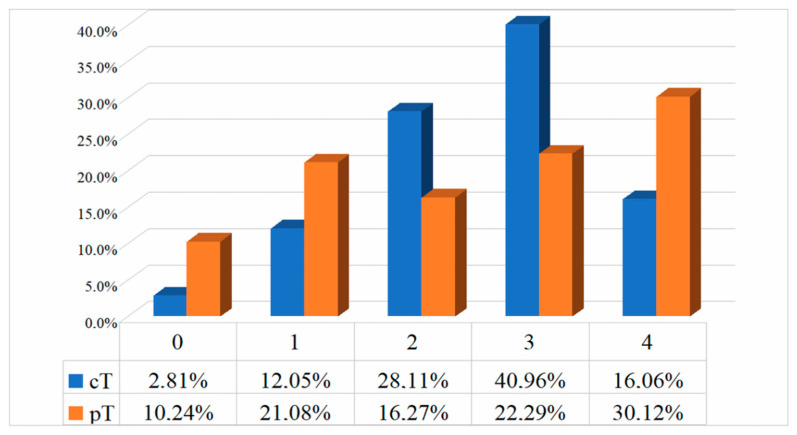
Differences between clinical (cT) and pathological (pT) staging of UCC.

Overall, the majority of patients were staged <cT2 disease (cT1–cT2—200 patients; 40.16%) or cT3 (204 patients; 40.96%) according to the post-chemotherapy CT scan (Figure 1). Based on post-surgical pathology, most patients were staged pT1–2 (186 patients; 37.35%) or pT4 (150; 30.12%; Figure 1).

**Figure 2:**
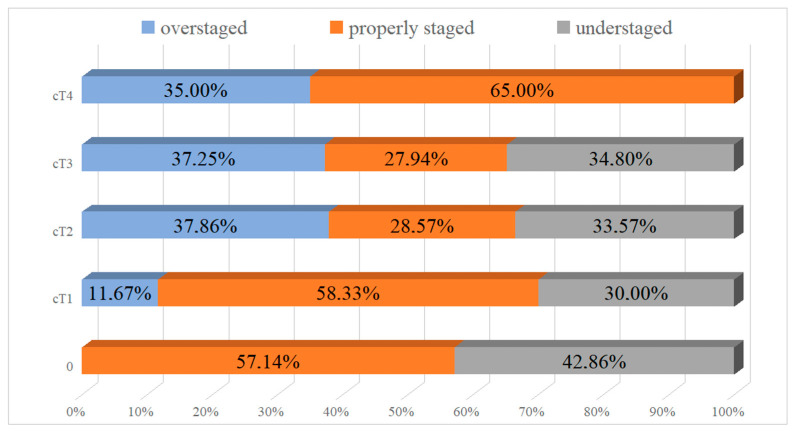
Over- and under-staging of disease according to the clinical cTNM scale.

Correlation between clinical and pathological stage, not surprisingly, revealed, that a higher clinical disease stage correlated with a higher pathological stage (based on the chi-squared test; *p* < 0.00005). However, in 306 patients (61.4%), the clinical stage did not agree with the pathological: in 142 patients (28.5%), the cancer was under-staged, and in 164 patients (32.9%), it was over-staged. Among patients staged cT1–cT2, 270 (54.2%) were incorrectly staged: 137 (27.5%) were under-staged and 133 (26.7%) were over-staged. More than half (8 out of 14, 57.14%) of patients with clinical stage cT0 were correctly diagnosed, compared to only a third of patients staged cT2 (39 out of 139 patients, 28%)) or cT3 (57 out of 204 patients, 27.9%) (Figure 2).

Status of the lymph nodes was also assessed in order to compare clinical vs. pathological status. Upon pathology, cancer cells were found in 19.36% of patients who were not suspected of metastasis during clinical lymph node staging. Clinical stage cN0 was in concordance with pathological pN0 in 80.64%, but 19.36% of patients assumed as N0 had nodal metastasis (10.93% were pN1, 7.74% pN2, and 0.68% pN3). On the other hand, 60% of patients with clinical stage cN3 had no metastasis in reality (pN0) (Table 2).

It was also examined whether UCC could spread to the lymph nodes in patients with a clinically low risk and localized disease. We confirmed that a higher clinical disease stage had a higher risk of metastasis (*p* = 0.0673, Pearson chi-squared test; *p* = 0.0130, Mann-Whitney test). In particular, patients with cT4 (high grade) disease had a 28.75% risk of the tumor spreading beyond the urinary bladder. However, patients with clinically assessed low and localized disease (cT0 and cT1) also had significant risk of metastasis (7.14% and 6.67% respectively).

In the studied group it was also established that initial diagnosis of hydronephrosis significantly increases the risk of having a higher pathological cancer stage (*p* < 0.000005). In patients with hydronephrosis, the majority (45.13%) had pT4 cancer, compared to only 20.12% of those without hydronephrosis. In addition, there were twice as many lymph node metastases found in patients with hydronephrosis than in those without (*p* = 0.0028; Table 3).

## 4. Discussion

UCC is a heterogeneous disease with multiple possible treatment options, ranging from surveillance through to transurethral procedures or radical cystectomy with neoadjuvant or adjuvant chemotherapy [10]. As radical cystectomy is associated with a high risk of developing perioperative complications (including death), proper patient qualification via accurate preoperative staging is crucial. Unfortunately, even with the use of modern imagining techniques, clinical evaluation of the extent of the disease in UCC remains challenging. Indeed, studies have found that almost half of patients estimated to have a T1 disease by clinical staging are under-staged [11,12,13]. Although both CT and MRI can be used for clinical staging, they show insufficient specificity in distinguishing organ-confined (cT2) disease from locally-invasive disease (cT3a) [14]. However, newer 3T MRI devices can achieve 91% sensitivity and 96% specificity when distinguishing superficial from invasive organ-confined disease (cT1–cT2), which is far better than the results obtained with CT [15]. In contrast, both MRI and CT show similar accuracy in detecting invasion of the adjacent organs and distant metastasis (including lymph node metastasis) [16].

Only about half of the UCC patients with a clinical diagnosis of cT1 disease were correctly diagnosed in our study, and even fewer (~30%) cT2 or cT3 patients were staged appropriately. This result was not surprising since both CT and MRI have a significant diagnostic bias in clinical practice [6]. Therefore, new modalities are needed to improve clinical staging. For example, 18F-fluorodeoxyglucose (FDG)-positron emission tomography (PET)/CT, or the multiparametric MRI (mpMRI) technique with a new VI-RADS scale might be valid options for improving clinical staging [17,18,19]. However, these more modern methods require further validation before they can routinely be used in the clinic. Furthermore, as each imaging modality is highly operator-dependent [20], the imaging results should be evaluated together with the results of histopathologic investigations taken during TURBT procedure (low grade vs. high grade cancer) to predict a patient’s preoperative cancer status.

The presence of hydronephrosis during the initial cancer diagnosis increases the risk of a high stage of cancer. Indeed, we found that most patients with hydronephrosis had pT4 disease. Similarly, patients with hydronephrosis had almost double the number of lymph node metastases than those without hydronephrosis (*p* = 0.0028). Therefore, patients with UCC who are also diagnosed with hydronephrosis should be considered for more radical treatment, even if imaging examinations show no evidence of peri-vesical invasion.

Positive surgical margins and lymph node status may play a role in the survival rates of patients with UCC who undergo radical cystectomy. Although the optimal extent of lymph node dissection (LND) is still not established, it is generally confirmed that typical LND performed during radical cystectomy should consist of removal of nodes cranially to the common iliac bifurcation, the internal iliac, presacral, obturator, and external iliac nodes. Indeed, survival rates are improved in patients who have a higher number of lymph nodes dissected [21], although it was recently proposed that the pattern of LND is more important than the total nodal count [22]. In addition, the patient’s nodal status plays a crucial role in deciding whether or not to administer adjuvant chemotherapy.

We found that when enlarged lymph nodes were present on CT scans, more than half were staged correctly, and cancer cells were present in the histopathologic evaluation. On the other hand, 10% of patients without enlarged lymph nodes on CT were found with nodal metastasis in the final pathological report. This discrepancy is mainly due to the inaccuracy of CT staging, which is primarily based on the size of lymph nodes, and its operator-dependent nature. Moreover, some information on nodal status can be drawn from the clinical extent of the tumor observed before surgery. In patients with cT3 and cT4 disease, almost 25% were diagnosed with lymph node metastasis upon histopathological evaluation.

Although patients staged as cT1 are generally good candidates for conservative treatment, there is a growing body of evidence that a considerable proportion of these patients are harboring nodal metastasis (in our study, this was 6.67%). Similarly, Wiesner et al. [23] reported that 6–15% of patients with non-muscle invasive bladder cancer had nodal metastasis. Moreover, when grouping together cT1 and cT2 disease, we found that almost 13% of patients with such a staging were diagnosed with nodal spread after surgery. Therefore, such patients should always be informed about the substantial risk of metastasis and the option of radical treatment.

Since a plethora of UCC patients are under-staged or over-staged, they are likely to be undergoing the incorrect treatment. This inaccuracy in staging may explain why the mortality rates remain high for advanced UCC despite promising recent therapeutic advances, such as cisplatin-based chemotherapy or immunotherapy. Indeed, even the most sophisticated chemotherapy is not helpful if the clinical stage of the disease is not properly established. Furthermore, new imaging modalities are required to distinguish cT1 and cT2 disease accurately.

As can be seen from results of our study, clinical evaluation of disease stage, despite modern techniques, is still a significant dilemma. There is no one single modality which can distinguish with acceptable certainty local, locally advanced and disseminated disease. Therefore, results of our treatment cannot be easily predicted and correct treatment cannot be applied to the correct patients in the correct time. A personalized approach to every single patient, combining a histopathologic report, with results of imagining studies and clinical evaluation, can help to identify patients who require radical oncological treatment. Possibly, new findings in the histology of the disease (luminal versus basal type of cancer) which adhere to the correct chemotherapy regime, together with new modalities in imaging, can aid in improving outcomes for radical cystectomy. Until then, we are in a difficult position, with a remarkable number of patients being over- and under-staged, and thus treated in a suboptimal manner.

There are some inherent limitations in the present study due to its retrospective nature. In addition, for logistical reasons, no central pathology review was performed; therefore, the number of analyzed lymph nodes was not adequately studied. Furthermore, because of its multi-center design, the study may have suffered from a lack of homogeneity of medical treatment, including surgery.

## 5. Conclusions

In conclusion, the clinical evaluation of the extent of the spread of UCC remains difficult. Our study suggests that a multimodal approach to staging may be helpful, specifically, by combining a histopathological report with imaging results. If a low-grade disease is found in histology, infiltration of the bladder wall and distant metastasis are less probable than in those with high-grade disease. A confirmation of hydronephrosis or enlarged lymph nodes on ultrasound provides further evidence of a high local disease stage. Finally, correct staging with multiple imaging techniques which prove nodal or distant metastasis is warranted. Such a multimodal approach would eliminate the bias of a single method.

## Figures and Tables

**Table 1:** Basic clinical–pathological data of study group.

Number of Patients (Males/Females)	Mean Values						
	Age (Years)	Number of Lymphnodes Removed	Positive Soft Tissue Surgical Margins	Hb (g/dL)	Creatinine (mg/dL)	BMI (kg/m^2^)	Albumin (g/dL)
533 (355/178)	68	11	48 (9%)	12.6	1.24	26.5	7.14

**Table 2:** Clinical versus pathological status of lymph nodes.

Clinical Stage of Nodes (cN)	Pathological Stage of Nodes (pN)			
	pN0	pN1	pN2	pN3
cN0	354 (80.64%)	48 (10.93%)	34 (7.74%)	3 (0.68%)
cN1	24 (68.57%)	4 (11.43%)	6 (17.14%)	1 (2.86%)
cN2	10 (52.63%)	4 (21.05%)	5 (26.32%)	0
cN3	3 (60.00%)	0	2 (40.00%)	0

**Table 3:** Diagnosis of hydronephrosis according to local invasion of cancer (pTNM).

Hydronephrosis	pTNM					Ʃ
	0	1	2	3	4	
Yes	43	88	63	68	66	328
	13.11%	26.83%	19.21%	20.30%	20.12%	100%
No	14	19	22	52	88	195
	7.18%	9.74%	11.28%	26.67%	45.13%	100%
Ʃ	57	107	85	120	154	523
	10.90%	20.46%	16.25%	22.94%	29.45%	100%

TNM: Tumor Nodes Metastasis; pTNM: Patients are staged undergo post-surgical histopathological estimation.

## Data Availability

All data supporting reported results can sent by mail upon the request.
